# Socio-economic and proximate determinants of under-five mortality in Guinea

**DOI:** 10.1371/journal.pone.0267700

**Published:** 2022-05-05

**Authors:** Bright Opoku Ahinkorah, Eugene Budu, Abdul-Aziz Seidu, Ebenezer Agbaglo, Collins Adu, Dorothy Osei, Aduragbemi Banke-Thomas, Sanni Yaya

**Affiliations:** 1 School of Public Health, Faculty of Health, University of Technology Sydney, Ultimo, Australia; 2 Korle Bu Teaching Hospital, Accra, Ghana; 3 Department of Real Estate Management, Faculty of Built and Natural Environment, Takoradi Technical University, Takoradi, Ghana; 4 Centre for Gender and Advocacy, Takoradi Technical University, Takoradi, Ghana; 5 College of Public Health, Medical and Veterinary Sciences, James Cook University, Townsville, Queensland, Australia; 6 Department of English, University of Cape Coast, Cape Coast, Ghana; 7 Department of Health Promotion, Education and Disability, Kwame Nkrumah University of Science and Technology, Kumasi, Ghana; 8 School of Human Sciences, University of Greenwich, London, United Kingdom; 9 LSE Health, London School of Economics and Political Science, London, United Kingdom; 10 School of International Development and Global Studies, University of Ottawa, Ottawa, Canada; 11 The George Institute for Global Health, The University of Oxford, Oxford, United Kingdom; Charite Universitatsmedizin Berlin, GERMANY

## Abstract

**Background:**

The death of children under-five years is one of the critical issues in public health and improving child survival continues to be a matter of urgent concern. In this paper, we assessed the proximate and socio-economics determinants of child mortality in Guinea.

**Methods:**

Using the 2018 Guinea Demographic and Health Survey (GDHS), we extracted demographic and mortality data of 4,400 children under-five years. Both descriptive and multivariable logistic regression analyses were conducted.

**Results:**

Under-five mortality was 111 deaths per 1,000 live births in Guinea. The likelihood of death was higher among children born to mothers who belong to other religions compared to Christians (aOR = 2.86, 95% CI: 1.10–7.41), smaller than average children compared to larger than average children (aOR = 1.97, 95% CI: 1.28–3.04) and those whose mothers had no postnatal check-up visits after delivery (aOR = 1.72, 95% CI: 1.13–2.63). Conversely, the odds of death in children with 2–3 birth rank & >2 years of birth interval compared to ≥4 birth rank and ≤2 years of birth interval were low (aOR = 0.53, 95% CI: 0.34–0.83).

**Conclusion:**

We found that household/individual-level socioeconomic and proximate factors predict under-five mortality in Guinea. With just about a decade left to the 2030 deadline of the Sustainable Development Goals (SDGs), concerted efforts across all key stakeholders, including government and development partners, need to be geared towards implementing interventions that target these predictors.

## Introduction

Globally, the death of children under-five years is one of the critical issues in public health and improving child survival continues to be a matter of urgent concern [1]. A renewed commitment to realize any such improvements is enshrined in the Sustainable Development Goal (SDG) 3, which has as one of its targets to ensure a reduction of under-five mortality to 25 deaths per 1000 live births globally by 2030 [2]. However, under-five mortality remains alarmingly high in many low- and middle-income countries (LMICs) [3]. In 2018 alone, an estimated 5.3 million children under-five years died globally, with sub-Saharan Africa (SSA) contributing more than half of the death burden (2.7 million) [4, 5]. At an average of 78 deaths of children under-five per 1,000 live births, one in every 13 children dies before their fifth birthday in the sub-Saharan African region. This prevalence is 16 times higher than the risk of children born in high-income countries [6]. Though significant progress has been made in Guinea, a 2019 UNICEF report found Guinea to be one of the countries with high under-five mortality rates, with the current rate at 101 deaths per 1,000 live births [6]. Intrapartum related complications, preterm birth complications, pneumonia, diarrhea, and malaria often contribute to the majority of such deaths [3, 6].

Efforts to reduce under-five mortality in any strategic manner require a clear understanding of its predictors. Several studies have been published concerning the predictors of under-five mortality in the sub-Saharan African region as a whole [7–10] and specific countries such as Ethiopia [11], Ghana [12], Sierra Leone [13], and Nigeria [14]. These studies have to varying degrees of significance, identified maternal age, birth weight, maternal education, wealth index, educational status and birth order, sex of the child, and geographical location as predictors of under-five mortality. This suggests that the findings of these studies conducted in other sub-Saharan African countries may not be particularly applicable in the context of Guinea. Moreover, each country in SSA may have a different socio-cultural context [12] and children in each of these countries may be exposed to different risks that can result in death. It is against this background that the present study investigated the proximate and socio-economic determinants of under-five mortality in Guinea. We believe that country-specific findings from this study could contribute to identifying critical priority interventions to address under-five mortality in Guinea.

## Methods

### Data source

The 2018 Guinea Demographic and Health Survey provided the data for this study (GDHS). Specifically, the children’s file, which contains data of children born five years prior to the survey, was used. GDHS is part of several surveys obtainable from the MEASURE DHS Program, which contains information on a number of issues on population, health, and nutrition, including under-five mortality. Data for 4,400 children under the age of five, who served as the study’s unit of analysis, were gathered by interviewing women who had given birth within the previous five years. Due to the inclusion of variables that asked for information about the partners of women with children under-five years, the sample size of 4400 included only children born to mothers who were either married or cohabiting at the time of the study. The GDHS utilized a multi-stage, stratified sampling design to select all eligible women for interviews from households, which were considered sampling units [15]. The dataset if freely available for download at https://dhsprogram.com/data/dataset/Guinea_Standard-DHS_2018.cfm?flag=0

### Conceptual framework

**Fig 1:**
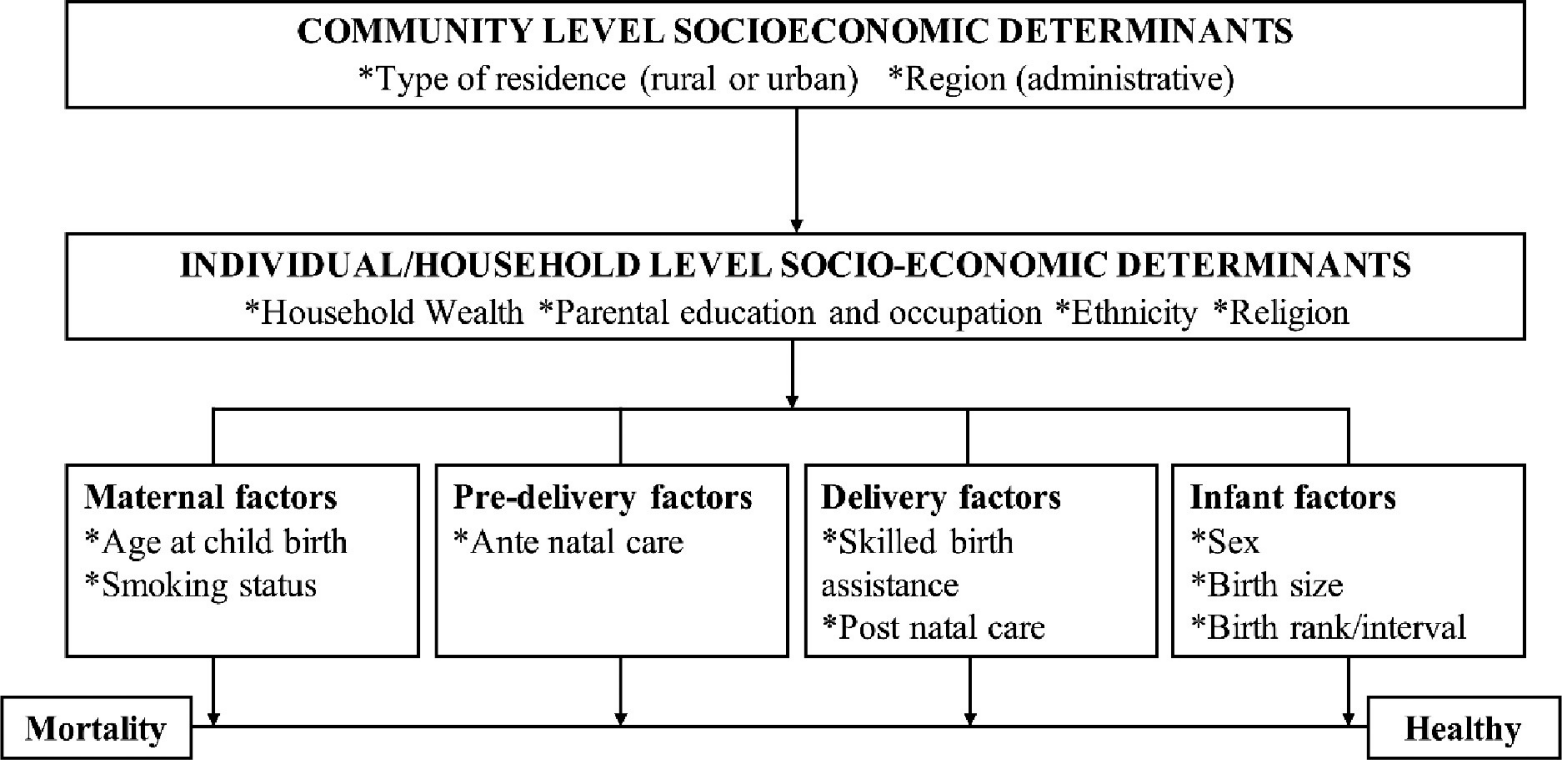
Conceptual framework of determinants influencing under-five mortality. Source: Mosley and Chen [16].

Based on the variables available in the 2018 GDHS datasets and earlier research [9–14], the study’s conceptual framework was developed from Mosley and Chen’s conceptual framework for the study of child survival in developing countries (Fig 1) [16].

### Study variables

The outcome variable was under-five mortality, which is the death of a child under five years (0–59 months) of life. To derive this variable, responses on whether the child under-five is alive or dead was recoded into binary outcome (0 = No and 1 = Yes). The variable was estimated based on complete maternal birth histories that include the date of every live birth (singleton and multiple births), survival status, current age for living children and age at death of children [9–14]. The explanatory variables considered in this study included two community-level socioeconomic variables (place of residence and region), seven household and individual level socioeconomic variables (wealth index, maternal ethnicity, maternal religion, maternal and paternal highest level of education, and maternal and paternal occupation) and nine proximate determinants variables (sex of child, birth size, birth rank and birth interval, age of mother at childbirth, antenatal care visit, use of tobacco, place of delivery, delivery assistance, and postnatal check-up (PNC) visits.

### Data analysis

The first step of the analysis involved the use of frequency tabulations to describe the proportions of all the explanatory variables, followed by a distribution of under-five mortality per the explanatory variables considered in this study, with their respective confidence intervals (CIs). We used the STATA command “syncmrates”, which calculates child mortality rates using the synthetic cohort probability method employed in Demographic and Health Surveys (DHS) to calculate the under-five mortality rates. The under-five mortality rates were obtained using 7,885 children born in the last five years. However, for subsequent analyses, the unit was children born to married and cohabiting women (4,400) due to the inclusion of variables related to partners of mothers of children under-five. This was followed by the use of Pearson’s chi-square to test the association between the explanatory variables and under-five mortality. Statistical significance was pegged at p<0.20. A multicollinearity test was performed on all statistically significant variables prior to the multivariable hierarchical logistic regression analysis to check if there was evidence of multicollinearity between them. The results showed no evidence of multicollinearity except family size and mother’s age at childbirth. These were therefore excluded from the study. The Multicollinearity test was done using the variance inflation factor. Whether significant or not, all the explanatory variables in the chi-square test were included in a multivariable logistic regression analysis. The multivariable logistic regression analysis was carried out in three stages.

The first step of the multivariable hierarchical logistic regression analysis included community-level socioeconomic indicators and under-five mortality (see Model I). In the second step, household and individual-level socioeconomic characteristics were included in the first model to investigate their association with under-five mortality (see Model II). In the last step, the proximate determinants were added to Model II to analyze their relationship with under-five mortality. Pseudo R2 was used to measure the logistic models’ goodness-of-fit. Stata SE version 14.2 was used for data cleansing, management, and analysis (StataCorp, College Station, Texas, USA). We utilized sample weight (v005/1,000,000) to compensate for over and under-sampling and the svy command to account for the complicated survey design and generalizability of the findings.

### Ethical considerations

The actual conduct of the 2018 GDHS received ethical approval from the ICF’s ethical committee. All survey participants provided informed consent and permission to use the data anonymously, which the enumerators obtained [15]. We acquired permission from the DHS program to utilize the 2018 GDHS data in our analysis. There was no need for additional ethical approval because our study only comprised secondary data analysis of publically available data that did not contain any personal information linked to the actual survey participants.

## Results

This study includes a total of 4,400 children below five years. Table 1 indicates results on the characteristics and the distribution of under-five mortality. The majority of the children were from the Kankan region (18.9%). A greater proportion of the children were from rural areas (74.7%), middle poorest quintile households (25.4%), Peulh ethnic group (37.8%) and Muslims (88.1%). Close to 52% of the children were males, 36.7% were average in size, and 39.9% were born with a ≥4 birth rank & >2 years of birth interval.

**Table 1:** Under-five mortality rate (per 1,000 live births) and unadjusted odds ratio by explanatory variables (n = 4400, weighted).

Determinants	Frequency (n)	Percentage (%)	U5MR [95% CI]	p-values*
**Region**				0.022
Boke	482	11.0	126 [96–157]	
Conakry	422	9.6	37 [34–51]	
Faranah	487	11.1	136 [107–164]	
Kankan	831	18.9	134 [111–154]	
Kindia	681	15.5	124 [97–151]	
Labe	515	11.7	105 [77–133]	
Mamou	356	8.1	99 [73–126]	
N’zerekore	626	14.2	112 [76–149]	
**Type of place of residence**				0.004
Urban	1112	25.3	60 [49–71]	
Rural	3288	74.7	131 [119–143]	
**Wealth Index**				0.003
Poorest	1118	25.4	139 [119–160]	
Poorer	1027	23.3	143 [120–165]	
Middle	889	20.2	129 [102–155]	
Richer	798	18.1	81 [61–101]	
Richest	567	12.9	41 [26–56]	
**Ethnicity**				0.047
Soussou	808	18.4	110 [83–138]	
Peulh	1662	37.8	106 [91–120]	
Malinke	1415	32.2	119 [100–139]	
Kissi	209	4.8	115 [62–168]	
Toma	40	0.9	51 [15–87]	
Guerze	267	6.1	142 [88–195]	
**Religion**				<0.001
Christianity	421	9.6	110 [83–138]	
Islam	3921	89.1	111 [101–123]	
Others (Hinduism, Buddhism, Atheism, Juddaism, Taoism, Confucianism, Sikhism)	58	1.3	229 [97–360]	
**Highest educational level of mother**				0.009
No education	3510	79.8	121 [109–133]	
Primary	457	10.4	114 [81–148]	
Secondary /Higher	432	9.8	57 [39–75]	
**Highest educational level of partner**				0.174
No education	3302	75.0	124 [112–136]	
Primary	316	7.2	117 [87–146]	
Secondary/Higher	782	17.8	79 [63–196]	
**Mother’s Occupation**				0.002
Not working	1059	24.1	100 [84–116]	
Official	93	2.1	70 [14–125]	
Sales and services	1048	23.8	118 [96–140]	
Agricultural	1531	34.8	135 [120–151]	
Manual	670	15.2	84 [64–104]	
**Father’s Occupation**				0.160
Not working	248	5.6	84 [59–109]	
Official	309	7.0	75 [42–109]	
Sales and services	831	18.9	86 [67–104]	
Agricultural	2037	46.3	134 [119–150]	
Manual	975	22.2	127 [105–148]	
**Size of child at birth**				<0.001
Very large	1174	26.7	112 [81–143]	
Larger than average	1128	25.6	102 [85–119]	
Average	1615	36.7	112 [92–132]	
Smaller than average	305	6.9	180 [127–233]	
Very small	178	4.1	207 [149–264]	
**Sex of the child**				0.812
Male	2273	51.7	115 [103–127]	
Female	2127	48.3	110 [97–124]	
**Birth rank and birth interval**				0.004
First birth rank	675	15.4	97 [80–115]	
2–3 birth rank & ≤2 years of birth interval	250	5.7	120 [88–150]	
2–3 birth rank & >2 years of birth interval	1355	30.8		
≥4 birth rank & ≤2 years of birth interval	363	8.3	119 [106–132]	
≥4 birth rank & >2 years of birth interval	1757	39.9	94 [80–107]	
**Age of mother at childbirth**				0.290
<20 years	2816	64.0	121 [109–133]	
More than 20 years	1584	36.0	99 [82–116]	
**Antenatal care visit**				0.135
No	695	15.8	108 [76–140]	
Yes	3705	84.2	91 [73–109]	
**Use of tobacco**				0.443
No	4338	98.6	113 [102–124]	
Yes	61	1.4	110 [56–164]	
**Place of delivery**				0.010
Home	2234	50.8	139 [121–156]	
Health facility	2166	49.2	99 [82–116]	
**Delivery assistance**				0.003
By TBA/Others	1906	43.3	125 [109–140]	
By SBA/Health professional	2494	56.7	100 [83–117]	
**Postnatal check visits**				0.001
No	2746	62.4	136 [119–152]	
<24 hours	1587	36.1	74 [45–103]	
≥1 Day	67	1.5	116 [24–257]	
National total	4,400		111 [101–122]	

*p-values generated from Pearson’s chi-square test.

Under-five mortality was 111 deaths per 1,000 live births in the country. Deaths of children under five was 136 per 1,000 live births in the Faranah region compared to 37 deaths per 1,000 live births in the Conakry region. Similarly, under-five mortality was higher in the rural areas (131 deaths per 1,000 live births) compared to the urban areas (60 deaths per 1,000 live births). Children from poorer households experienced a higher under-five mortality rate (143 deaths per 1,000 live births) compared to those from richest households (41 deaths per 1,000 live births). Children who were very small at birth had a higher under-five mortality rate (207 deaths per 1,000 live births) than those with larger than average size at birth (102 deaths per 1,000 live births). Under-five mortality was high among children with 2–3 birth rank & ≤2 years of birth interval (120 deaths per 1,000 live births) compared to those with ≥4 birth rank & ≤2 years of birth interval (94 deaths per 1,000 live births). Regarding the sex of the child, under-five mortality was high among males (115 per 1,000 live births) compared to females (110 per 1,000 live births).

Table 2 shows the results of the multivariable logistic regression analysis. In model III, all the community, household/individual-level socioeconomic and proximate factors were included in the analysis. In this model, religion, size of the child at birth, birth rank and birth interval, and postnatal check-ups had significant associations with under-five mortality. The likelihood of death was higher among children born to mothers who belong to other religions compared to Christians (aOR = 2.86, 95% CI: 1.10–7.41), smaller than average children compared to larger than average children (aOR = 1.97, 95% CI: 1.28–3.04) and those whose mothers had no postnatal check-up visits after delivery compared to those whose mothers had postnatal check-up visits less than 24 hours after delivery (aOR = 1.72, 95% CI: 1.13–2.63). Conversely, there was lower odds of death in children with 2–3 birth rank & >2 years of birth interval compared to ≥4 birth rank and ≤2 years of birth interval (aOR = 0.53, 95% CI: 0.34–0.83).

**Table 2:** Multivariate hierarchical logistic regression results by determinants for under-five mortality -adjusted odds ratio.

Determinants	Model I			Model II			Model III		
**Region**	aOR			aOR	95% CI		aOR	95% CI	
Boke	1.47	0.69	3.16	1.29	0.59	2.80	1.21	0.55	2.67
Conakry	1			1			1		
Faranah	2.17*	1.01	4.97	1.78	0.80	3.99	1.78	0.79	4.03
Kankan	1.79	0.85	3.77	1.35	0.59	3.07	1.35	0.59	3.08
Kindia	1.77	0.85	3.73	1.59	0.74	3.38	1.53	0.71	3.28
Labe	1.26	0.56	2.85	1.34	0.55	3.38	1.29	0.52	3.20
Mamou	1.08	0.48	2.47	1.07	0.45	2.58	1.09	0.45	2.64
N’zerekore	1.78	0.81	3.90	1.11	0.45	2.75	1.17	0.47	2.92
**Type of place of residence**									
Urban	1			1			1		
Rural	1.44*	1.02	2.06	0.93	0.54	1.60	0.90	0.52	1.55
**Wealth Index**									
Poorest	-	-	-	2.34*	1.01	5.43	2.07	0.89	4.81
Poorer	-	-	-	2.33*	1.02	5.30	2.07	0.90	4.75
Middle	-	-	-	2.09	0.94	4.64	1.88	0.85	4.18
Richer	-	-	-	1.61	0.84	3.07	1.53	0.80	2.94
Richest				1			1		
**Ethnicity**									
Soussou	-	-	-	0.80	0.12	5.25	0.73	0.11	4.75
Peulh	-	-	-	0.74	0.12	4.65	0.64	0.10	4.00
Malinke	-	-	-	0.95	0.15	5.91	0.81	0.13	5.03
Kissi	-	-	-	0.59	0.14	3.02	0.55	0.11	2.87
Toma				1			1		
Guerze	-	-	-	1.47	0.31	6.94	1.38	0.29	6.67
**Religion**									
Christianity				1			1		
Islam	-	-	-	1.31	0.37	4.62	1.23	0.35	4.34
Others (Hinduism, Buddhism, Atheism, Juddaism, Taoism, Confucianism, Sikhism)	-	-	-	3.75**	1.46	9.62	2.86*	1.10	7.41
**Highest educational level of mother**									
No education	-	-	-	1.63	0.87	3.08	1.42	0.75	2.68
Primary	-	-	-	1.44	0.70	2.95	1.34	0.65	2.74
Secondary/Higher				1			1		
**Highest educational level of partner**									
No education	-	-	-	0.96	0.64	1.46	0.91	0.60	1.37
Primary	-	-	-	0.81	0.43	1.51	0.80	0.43	1.48
Secondary/Higher				1			1		
**Mother’s Occupation**									
Not working	-	-	-	0.75	0.22	2.58	0.72	0.21	2.50
Official				1			1		
Sales and services	-	-	-	1.31	0.38	4.49	1.29	0.37	4.47
Agricultural	-	-	-	0.91	0.27	3.11	0.85	0.24	2.92
Manual	-	-	-	0.67	0.19	2.37	0.67	0.19	2.40
**Father’s Occupation**									
Not working	-	-	-	0.89	0.38	2.04	0.82	0.36	1.89
Official				1			1		
Sales and services	-	-	-	1.07	0.55	2.07	1.12	0.60	2.13
Agricultural	-	-	-	1.04	0.54	2.01	1.04	0.55	1.96
Manual	-	-	-	1.13	0.59	2.16	1.19	0.63	2.22
**Size of child at birth**									
Very large	-	-	-	-	-	-	0.88	0.61	1.27
Larger than average							1		
Average	-	-	-	-	-	-	0.91	0.66	1.27
Smaller than average	-	-	-	-	-	-	1.97***	1.28	3.04
Very small	-	-	-	-	-	-	1.72	1.00	2.94
**Sex of the child**									
Male	-	-	-	-	-	-	0.98	0.76	1.26
Female							1		
**Birth rank and birth interval**									
First birth rank	-	-	-	-	-	-	0.63	0.38	1.05
2–3 birth rank & ≤2 years of birth interval	-	-	-	-	-	-	0.83	0.45	1.52
2–3 birth rank & >2 years of birth interval	-	-	-	-	-	-	0.53**	0.34	0.83
≥4 birth rank & ≤2 years of birth interval							1		
≥4 birth rank & >2 years of birth interval							0.74	0.50	1.11
**Age of mother at childbirth**									
<20 years	-	-	-	-	-	-	1.06	0.81	1.38
More than 20 years							1		
**Antenatal care visit**									
No	-	-	-	-	-	-	1.08	0.77	1.51
Yes							1		
**Use of tobacco**									
No	-	-	-	-	-	-	0.70	0.26	1.87
Yes							1		
**Place of delivery**									
Home							1		
Health facility	-	-	-	-	-	-	1.53	0.89	2.65
**Delivery assistance**									
By TBA/Others	-	-	-	-	-	-	1.26	0.77	2.06
By SBA/Health professional							1		
**Postnatal check of visits**									
No	-	-	-	-	-	-	1.72*	1.13	2.63
<24 hours							1		
≥1 Day	-	-	-	-	-	-	1.25	0.38	4.15

Exponentiated coefficients; 95% confidence intervals in brackets; aOR adjusted Odds Ratios CI Confidence Interval.

* *p*≤0.05

** *p*≤0.01

*** *p*≤0.001.

Model I adjusted for community level socioeconomic factors.

Model II adjusted for community and household/individual level socioeconomic factors.

Model III adjusted for community, household/individual level socioeconomic and proximate factors.

## Discussion

In this study, we investigated the determinants of under-five mortality in Guinea using data from the most recent DHS survey conducted in 2018. Our study revealed that region of residence, religion, birth order, birth interval, and postnatal check-up visits are strong predictors of under-five mortality in Guinea. These factors are discussed further in the subsequent paragraphs.

Our study also revealed a strong relationship between religion and under-five mortality in Guinea. Specifically, children born to Christians recorded a lower likelihood of under-five mortality compared to their counterparts whose mothers were practitioners of other religions (Hinduism, Buddhism, Atheism, Judaism, Taoism, Confucianism, Sikhism). A lower likelihood of under-five mortalities among Christians has been reported in Mozambique [17], Ghana [18], and Brazil [19]. With nearly 85 percent of Guinea’s population being Muslim, Islam is the demographically, socially, and culturally dominant religion. This study’s conclusion could be explained by prior research findings on the cultural practice of keeping newborns hidden from the public due to fear of harm after birth [20]. To reduce under-five mortality among religious groups in Guinea, religious leaders’ role in promoting maternal and child healthcare utilization must be increased. This study implies that the impact of religion on under-five mortality should be examined further to understand the underlying factors better.

Children born to poor women were more likely to die compared to those born to rich women. According to research, wealthier women are more likely to use maternal and childcare. [21]. Most poor women live in rural areas where 84% of babies in urban areas receive a postnatal examination, compared to only 43% of babies in rural areas. A similar disparity exists with regard to access to complete immunization and postnatal care [22]. Poor women are also characterized by a lack of essential services (such as potable water) and proper sanitation practices, the lack of which causes diarrhea and dehydration in children in rural Guinea, resulting in the death of many children in rural Guinea [23]. Similar observations have been made in Nigeria [24, 25], Kenya [26], Turkey [27], and Ethiopia [25]. Possible explanations include lower socio-economic status and households’ inability to cover childbirth costs [28].

Furthermore, birth rank and birth interval and birth size were also associated with under-five mortality among children in Guinea. In this regard, our study revealed that the odds of death in children with 2–3 birth rank & >2 years of birth interval compared to ≥4 birth rank and ≤2 years of birth interval. This finding agrees with those of some previous studies in Ethiopia [29], Ghana [30, 31], and Uganda [32]. Yaya et al. also made a similar observation in Chad, the Democratic Republic of Congo, Mali, Niger and Zimbabwe [33]. Children with smaller birth sizes were more likely to die before age five than those with very large sizes at birth. Akin to that, a previous study in India identified small birth size as a strong determinant for child mortality [34].

Children of parents who had no postnatal check-up visits also had higher risks of dying before their fifth birthday than those who had postnatal check-ups. This finding resonates with the findings of other studies [10, 35–37]. The social institutions of society, such as health facilities where women obtain PNC services, may expose mothers to helpful health information such as the benefits of using PNC services for their newborns, influencing their care for their babies. A plausible reason given in the literature is that access to healthcare services, including postnatal care, reduces the likelihood of children dying before their fifth birthday. In practice, reproductive healthcare services are provided in both publicly and privately owned health clinics and hospitals in Guinea. However, a study by Ettarh and Kimani did not find any significant association between postnatal check-up visits and under-five mortality [26].

### Strengths and limitations

The main strength of this study lies in its use of a nationally representative data. With this, the findings can be generalized to all children in Guinea. Notwithstanding this, the findings need to be interpreted in relation to some limitations. In the first place, in terms of causal relation between the variables measured, this study is handicapped due to the cross-sectional nature of the study design. Again, the data for the study is also subject to recall bias, as the parents may not be able to give accurate information because of the retrospective nature of reporting child deaths. Some important factors such as family size and age of mother at first birth were not included due to collinearity issues.

### Policy implications

The study’s findings serve as a starting point for further debate. Our findings have several policy implications. While there is a need to address the different variables across Guinea, our study findings imply that special attention should be paid to some specific groups who appear to be the most susceptible among the vulnerable. For starters, children in the country’s poorer districts, such as Farannah, will benefit from steps to reduce inequality. Furthermore, behavioral change communication about the importance of postnatal checks for babies, as well as family planning and spacing, must be implemented while ensuring that those efforts reach moms and dads of large families with a low birth rank and interval, as well as non-Christians. Furthermore, campaigning on the importance of postnatal check-ups after birth can be performed both during mothers’ interaction with the health system, such as during ante-natal care clinics and more generally to the broader community, as part of outreaches.

## Conclusion

We found that socioeconomic and proximal determinants are associated with under-five mortality. As a result, it is vital that interventions aimed at reducing under-five mortality take these factors into account. Future studies should focus on understanding the processes through which these factors contribute to childhood mortality. However, with less than a decade until the SDGs’ 2030 deadline, all major players, including the government and development partners, must work together to assist Guinea meet its under-five mortality reduction targets.

## Abbreviations

aOR: Adjusted Odds Ratio
CI: Confidence Interval
GDHS: Guinea Demographic and Health Survey
VIF: Variance Inflation Factor
PNC: postnatal check-up
SDG: Sustainable Development Goal
LMICs: Low-and Middle-Income Countries
